# A Contemporary Multifaceted Narrative Review on Thyroid Dysfunction in People Living with Human Immunodeficiency Virus

**DOI:** 10.3390/biomedicines13112613

**Published:** 2025-10-25

**Authors:** Mohanad Alhalabi, Mohamed M. Attian, Lana Alhalabi, Dushyant Mital, Omar Alhalabi, Mohamed H. Ahmed

**Affiliations:** 1 Faculty of Medicine and Health Sciences, University of Buckingham, Buckingham MK18 1EG, UK; 2109469@buckingham.ac.uk (M.A.); 2108480@buckingham.ac.uk (M.M.A.); 2 Department of Medicine, Royal Surrey County Hospital, Guildford GU2 7XX, UK; lana.alhalabi04@gmail.com; 3 Department of Blood Borne Viruses and HIV, Milton Keynes University Hospital NHS Foundation Trust, Eaglestone, Milton Keynes, Buckinghamshire MK6 5LD, UK; dushyant.mital@mkuh.nhs.uk; 4 Department of Medicine, Tunbridge Wells Hospital, Pembury TN2 4QJ, UK; omar.alhalabi1@nhs.net; 5 Department of Geriatric Medicine, Milton Keynes University Hospital NHS Foundation Trust, Eaglestone, Milton Keynes, Buckinghamshire MK6 5LD, UK; 6 Honorary Senior Lecturer of the Faculty of Medicine and Health Sciences, University of Buckingham, Buckingham MK18 1EG, UK; 7 Department of Medicine and HIV Metabolic Clinic, Milton Keynes University Hospital NHS Foundation Trust, Eaglestone, Milton Keynes, Buckinghamshire MK6 5LD, UK

**Keywords:** thyroid, HIV, hypothyroidism, hyperthyroidism and thyroid malignancy

## Abstract

The use of highly active combined antiretroviral therapy (cART) has increased life expectancy in people living with HIV (PLWH). As a result of ongoing monitoring and surveillance in established HIV out-patient clinics, thyroid dysfunction amongst this population has become increasingly reported. In this narrative review, primary studies, case reports, and meta-analyses published on PubMed, Embase, and Cochrane were analysed. The most reported thyroid dysfunction is subclinical hypothyroidism (SCH). The prevalence of subclinical hypothyroidism was as high as 40% in PLWH with CD4 T-cell count < 350 cells/mm^3^, which is a level indicating a state of immunosuppression. Some less commonly reported thyroid dysfunctional conditions include overt hyperthyroidism and thyroid malignancy. Reports have linked the development of thyroid dysfunction to the use of cART, leading to immune reconstitution inflammatory syndrome (IRIS), which has also been linked to the development of Grave’s disease (GD). It is also important to check for thyroid malignancy, as PLWH are prone to having a high risk of developing non-AIDS-related or -defining cancer (NADC). Most research suggests symptom-driven monitoring. However, evidence also suggests that monitoring with cART status change, monitoring for patients with significant comorbidities, or with immune reconstitution may be useful. The screening should include Free Thyroxine (FT4), triiodothyronine (FT3), and thyroid-stimulating hormone (TSH) testing. Furthermore, vigilance for Grave’s disease and performing thyroid antibody checks are advised, especially once the reconstitution of T-cells is achieved.

## 1. Introduction

Human Immunodeficiency Virus (HIV) is a retrovirus that targets CD4+ T-lymphocyte helper cells. As a result, CD4+ T-cell count reduces, resulting in extreme immune suppression. Subsequently, this makes PLWH susceptible to opportunistic infections [1].

HIV is a significant public health issue as it has affected 40.8 million people in 2024, including 1.4 million children [2]. Fortunately, the disease prognosis has improved due to the development of effective cART. This therapy has allowed HIV to be treated as a manageable chronic condition rather than a terminal disease that it once was [1]. However, despite these developments, there have been 630,000 HIV-related deaths globally, with 75,000 being children [2]. 

Furthermore, there has been increasingly more research on the link between HIV and autoimmune endocrine conditions. As PLWH live longer due to cART, they also develop co-morbidities. Of these co-morbidities, thyroid dysfunction has been shown to be more common in PLWH. The prevalence of thyroid dysfunction in PLWH varies between studies. An earlier study reported overt thyroid disease in 1–2% of PLWH and subtle abnormal thyroid function test finding in 35% of PLWH [3,4]. More recently, a meta-analysis also showed that PLWH are at risk of developing manifestations of thyroid abnormalities. These abnormalities affect the quality of life in these individuals. Evidence suggests that PLWH are at a higher risk of developing subclinical thyroid diseases, isolated low FT4 levels, and euthyroid sick syndrome. The development of subclinical thyroid disease has been associated with the progression of overt thyroid disease in PLWH [5]. Consequently, overt hypothyroid disease can lead to complications including neuropathy, impairment in cognitive function, infertility, cochlear dysfunction, and dysregulation in glucose metabolism. It can also affect the cardiovascular system by increasing vascular resistance and decreasing left ventricular function and cardiac output [6]. In addition, overt hyperthyroid disease can lead to ophthalmopathy, dermopathy, and cardiovascular complications as well [7].

In this narrative review, we will summarise the current research on the relationship between PLWH and thyroid disease. We also aim to address the cost-effectiveness of thyroid disease screening for this cohort. This study is a narrative review that collects and presents current research on HIV and thyroid function. A structured literature search was conducted using electronic databases including PubMed (MEDLINE), Embase, and Cochrane Library. The search strategy included a combination of Medical Subject Headings (MeSH) terms and free text terms to capture all possible relevant studies. Search terms are listed below. The search covered all the literature published on the relationship between thyroid dysfunction and HIV.

Search terms: Hypothyroidism, hyperthyroidism, Grave’s disease, Thyroiditis, TSH, Papillary thyroid cancer, thyroid nodule, thyroid cancer, medullary thyroid cancer, HIV, Antiretroviral therapy, Highly active antiretroviral therapy, AIDS, Immune reconstitution inflammatory syndrome, combination antiretroviral therapy, and PLWH.

## 2. Prevalence of Subclinical Hypothyroidism (SCH) in HIV-Infected Populations

**Figure 1:**
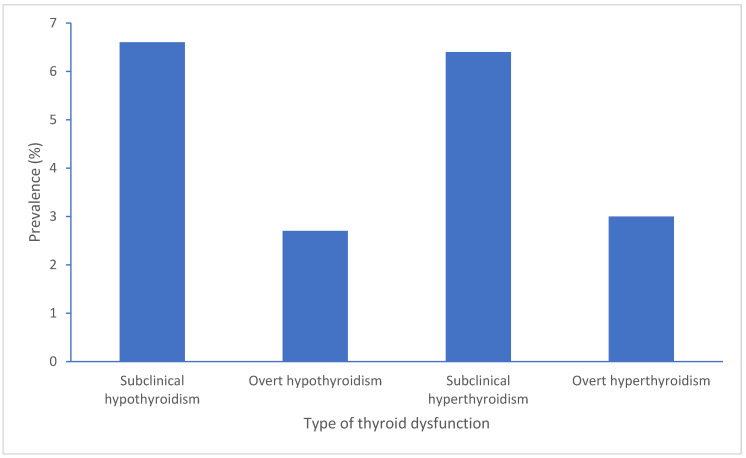
Summary of prevalence range reports on different thyroid dysfunctions in PLWH.

The condition of SCH presents as increased TSH levels combined with normal FT4 levels in PLWH where SCH links to immune conditions along with HIV disease management and therapy approaches especially with cART treatment strategies [8]. Early detection becomes crucial because SCH exists with elevated TSH levels and normal free T3 and T4 levels, although patients might experience no symptoms or display vague symptoms and signs [9]. The condition presents as elevated TSH values together with FT4 and FT3 levels remaining in the normal ranges according to TSH reference values set between 0.4 and 4.0 μIU/mL [10,11]. SCH requires a TSH measurement above the normal range but less than 10 μIU/mL to be diagnosed [11]. Most cases remain without apparent symptoms, and thus, screening tests may be useful for detection [9,12,13]. One study estimated SCH prevalence in males living with HIV to be 6.6%, and another study on Spanish population estimated SCH prevalence to be 3.5% [8]. HIV itself per se appears to cause thyroid dysregulation because of persistent inflammation and immune system problems [8]. Lower CD4 counts have been linked to SCH occurrence, and research shows that SCH risk increases with decreasing CD4 cell counts [8,14]. A meta-analysis looking at patients with HIV estimated SCH prevalence to be 7.7% [5]. In a prevalence study involving 350 Indian children with HIV infection, 10% were found to have subclinical hypothyroidism while also showing connections to reduced CD4 counts and compromised immune status [15]. The start of cART can trigger thyroid dysfunctions including SCH in patients [15]. The prevalence of SCH proves higher in specific populations compared to HIV-negative controls and individuals who have not received cART (Naïve patients) [16]. PLWH display substantially higher thyroid dysfunction rates compared to the general population which ranges between 4% and 10%, with some patients reaching 40% prevalence when CD4 counts fall below 350 cells/mm^3^ [12,13]. Importantly, thyroid dysfunction affected 31.33% of participants with advanced immunosuppression leading to SCH [17]. A study from South Carolina reported a 16.9% prevalence of hypothyroidism among people living with HIV between 2005 and 2016 [18] The prevalence of SCH in India was shown to be 14.76% with HIV-driven immune destruction likely playing a role in thyroid dysfunction [13] (Figure 1).

Research studies have documented that subclinical hypothyroidism affects 3% to 12% of HIV patients undergoing cART treatment especially when their CD4 cell count remains low. The 10% prevalence of subclinical hypothyroidism in HIV-positive children requires prompt detection because it leads to growth problems and metabolic issues. The condition of subclinical hypothyroidism occurs mainly in patients with severe immune system suppression, and it indicates the beginning of thyroid organ failure that will become overt if left untreated. The prevalence of this condition remains higher among patients who receive stavudine-based treatment compared to those taking lamivudine or zidovudine [4,19,20].

The reported prevalence of SCH in PLWH shows significant variations between studies because of different study populations, geographic locations, demographic characteristics, cART exposure levels, immunosuppression intensity, diagnostic threshold, and laboratory methods. The existing differences between studies demonstrate the requirement for standardised diagnostic criteria and extended longitudinal research to establish the actual SCH prevalence in HIV patients and the frequency of monitoring.

## 3. Pathophysiology, Mechanisms, and Management of SCH

PLWH develop SCH due to immune activation that triggers autoimmune thyroid gland damage after cART-induced immune recovery [21], and CD4 count reductions correlate with TSH elevation and hypothalamic–pituitary–thyroid axis dysregulation [13]. The combination of chronic HIV infection with autoimmune responses produces thyroid dysfunction [12], while inflammatory cytokine damage to the hypothalamic–pituitary–thyroid axis elevates TSH without immediately affecting FT4 levels. cART therapy produces different effects on thyroid function because it stabilises the immune system. Additionally, thyroid hormone fluctuations can occur during initial treatment phases because of IRIS [22].

Some mechanisms contributing to thyroid damage includes opportunistic infections. For example, Pneumocystis jiroveci, Mycobacterium tuberculosis, Cryptococcus neoformans, and Coccidioides immitis infections can invade thyroid tissue to cause destructive thyroiditis. The opportunistic infection medications rifampin and isoniazid speed up thyroid hormone elimination through their effect on liver enzyme activity. The body’s ability to convert T4 to T3 through deiodinase enzymes becomes impaired when patients experience malnutrition or selenium deficiency which reduces thyroid hormone availability [20,23,24].

### 3.1. Impact of cART Regarding SCH

Studies demonstrate that cART treatment decreases TSH values in the long run, but cART initiation seems to boost the risk of SCH. Stavudine and lamivudine are two medications which have been linked to elevated TSH and SCH levels, but the former drug is not used anymore [25]. Regular thyroid monitoring may be useful in patients having cART therapy because SCH occurs more frequently in these patients [25]. After cART initiation, patients may experience IRIS which is linked to autoimmune thyroid dysfunction [8,25]. In addition, thyroid dysfunction occurs more frequently in cART-treated patients than in cART-naïve patients (39.4% vs. 24.3%) [26]. The duration of cART exposure has been associated with worsening thyroid function [10,16,27].

The World Health Organisation has recommended against stavudine use but other antiretrovirals including protease inhibitors like lopinavir/ritonavir can affect levothyroxine absorption and thyroid hormone metabolism. The need for regular TSH tests exists because of drug interactions that occur when patients change their treatment plan. The immune system’s recovery process after cART initiation leads to subclinical hypothyroidism, which appears quickly in patients who experience fast immune system recovery [20,28,29].

### 3.2. Clinical Features and Significance of SCH

SCH patients typically display non-differentiating symptoms including fatigue and weight fluctuations alongside depression which affect their life quality [21,30]. Levothyroxine therapy should be considered for patients with significantly elevated TSH or concerning symptoms because it can help improve their health status [21,31]. SCH has the potential to result in adverse health effects such as cognitive impairment, cardiovascular disease, weight changes, and hypothyroidism progression unless patients receive proper monitoring [10,16,18]. The presence of SCH symptoms negatively affects patient adherence to cART, leading to worse outcomes and reduced patient participation in monitoring [9].

The condition of subclinical and overt hypothyroidism produces specific effects on body organs which include peripheral neuropathy, cochlear dysfunction, and cognitive decline. The cardiovascular system experiences three main effects from SCH which include elevated vascular resistance, diastolic heart dysfunction, and abnormal lipid profiles leading to increased heart disease probability. The research indicates that SCH elevates the risk of myocardial infarction and accelerates atherosclerosis development by 1.5 to 2 times in HIV-positive patients [20,32,33].

### 3.3. SCH Screening Recommendations in PLWH

Although routine thyroid screening with TSH and FT4 has been suggested, the supporting evidence is limited. One small study reported an increased prevalence in subclinical hypothyroidism among PLWH receiving cART and recommended routine screening, albeit without specifying an interval [8]. On another note, a case report suggested a symptom-driven approach to testing, particularly in patients with elevated serum creatinine [25]. A cross-sectional study looking at patients from one centre recommends baseline TSH screening [16]. A paper published in the European Journal of Cardiovascular Medicine recommends obtaining TFTs after ART initiation especially when switching [34]. A study on 83 children living with HIV suggest annual thyroid function monitoring in such populations, especially those with significant immunosuppression [12].

### 3.4. Diagnosis and Management

According to the diagnosis thresholds, a TSH measurement between 4.5 and 10 mIU/L indicates SCH based on laboratory reference standards [18]. Treatment decisions should be based on clinical scenarios [9,13,17]. The management plan for patients with thyroid hormone deficiency depends on their TSH levels and symptoms, while those who are symptom-free can receive conservative monitoring [10,13,16,35]. Management approaches should be personalised to symptoms, CD4 counts, and comorbid conditions.

### 3.5. Patient Education and Follow-Up

Patients who receive updated information and education about SCH risks and regular monitoring benefits will show better adherence to their treatment plan [13,17]. Thus, thyroid function tests and appropriate care should form essential components of HIV treatment protocols.

## 4. Overt Hypothyroidism

The main endocrine disorder of overt hypothyroidism occurs when thyroid hormone production becomes insufficient, thus, resulting in high TSH and decreased FT4 levels. PLWH develop overt hypothyroidism more often than general population members, thus, requiring special attention in their care and health maintenance [8]. The condition affects PLWH on cART which is an important medical concern [8]. Overt hypothyroidism shows up as thyroid-stimulating hormone elevation together with decreased FT3 and FT4 levels which produce metabolic changes along with fatigue and diminished overall health [15,16].

The prevalence of overt hypothyroidism remains lower than subclinical hypothyroidism yet produces more significant health effects. The prevalence of overt hypothyroidism affects 2.5% to 3% of HIV patients, while patients with CD4 counts under 200 cells/mm^3^ show increased risk. The development of hypothyroidism occurs at different times relative to ART start and results from autoimmune thyroiditis, opportunistic infections, and cART toxicity [20,36,37].

### 4.1. Prevalence of Overt Hypothyroidism in PLWH

The research indicates that overt hypothyroidism affects 2.5% to 2.7% of PLWH, thus, indicating a health problem in this group [8]. Studies suggest that HIV infection produces thyroid dysfunction at rates exceeding the public population. This difference may be because of low CD4 count increasing the likelihood of hypothyroidism [8]. The condition remains frequently undiagnosed because it shares symptoms with HIV-related diseases [8]. Overt hypothyroidism also developed in a 10-year-old girl with perinatally acquired HIV while she was on long-term cART treatment [15]. According to research conducted in South Africa, thyroid dysfunction occurred in 55% of HIV-positive individuals, with overt hypothyroidism being most prevalent among patients taking cART [10]. The prevalence of overt hypothyroidism ranges between 0% and 2.6% according to various studies. This demonstrates hypothyroidism’s clinical importance although it occurs less frequently than other thyroid issues [10,13].

### 4.2. Mechanisms Involved in Overt Hyperthyroidism

The development of overt hypothyroidism in HIV patients involves various contributing factors [8]. HIV itself creates immunosuppression which makes patients more susceptible to autoimmune thyroiditis, thus, causing primary hypothyroidism [8]. cART initiation may result in IRIS which causes autoimmune thyroiditis that can result in overt hypothyroidism [8,15,27]. Patients with lower CD4 counts showing elevated TSH levels suggest that immunosuppression increases thyroid dysfunction risks [8,25]. Research suggests that HIV itself independently impacts thyroid function through inflammatory processes and disrupted immune responses, although the virus’s direct thyroid effects remain uncertain [10]. The patho-genetic mechanism of chronic HIV infection leads to hypothyroidism through distinct immune, infectious, and treatment-related processes. Systemic inflammation that persists for long periods causes IL-1, IL-6, and TNF-α to rise, which suppresses TRH production in the hypothalamus and TSH release from the pituitary gland and damages peripheral deiodinase enzymes, leading to hypothyroidism [38]. The thyroid gland becomes directly infected by Mycobacterium tuberculosis and cytomegalovirus and Cryptococcus which results in primary hypothyroidism through tissue destruction [39]. The combination of hepatitis C infection with other diseases leads to immune tolerance breakdown which produces more thyroid antibodies and increases the risk of autoimmune thyroiditis resulting in hypothyroidism [40]. The process of immune system recovery through cART treatment sometimes reveals hidden autoimmune thyroiditis which causes hypothyroidism, and specific antiretroviral medications have shown inconsistent links to thyroid hormone imbalances [28]. cART helps restore immune function while affecting the risk of autoimmune thyroid diseases specifically in patients who had low nadir CD4 counts [10,13,41].

The development of hypothyroidism in HIV patients is influenced by multiple factors beyond ART treatment including direct thyroid cancer infiltration from Kaposi sarcoma and lymphoma which occur more frequently in patients with advanced HIV. The anti-infective medication rifampin accelerates T4 metabolism, which can lead to worsening hypothyroidism. The combination of selenium and iodine deficiencies with deiodinase activity reduction leads to increased hormonal deficits. The multiple factors that contribute to hypothyroidism in HIV patients interact with cART and immune system problems to create a complex set of causes [20,42,43].

### 4.3. Impact of ART on Development of Overt Hypothyroidism

cART serves as a vital treatment for HIV, yet research shows that it is associated with overt hypothyroidism [8,25]. Research indicates that stavudine and lamivudine together with nevirapine have been associated with thyroid problems by modifying thyroid hormone levels or altering the immune response [8,25,44]. cART-related immune system recovery can trigger IRIS, leading to autoimmune thyroid diseases which often result in more severe hypothyroidism [25]. The research demonstrates that TSH levels decrease as CD4 counts improve, yet endocrine complications continue to exist [5,10].

The strongest connection with both overt and subclinical hypothyroidism is with stavudine, while zidovudine and lamivudine show a weaker association. Furthermore, protease inhibitors can block levothyroxine absorption which decreases treatment effectiveness. The World Health Organisation has suggested stopping stavudine treatment because of its adverse effects on metabolism and endocrine system, but this drug continues to be used in certain resource-limited areas. The specific drug-related effects on thyroid function require healthcare providers to modify ART treatment plans according to thyroid test results [19,20,45].

### 4.4. Clinical Features and Significance for Overt Hypothyroidism

Overt hypothyroidism in patients creates similar symptoms to HIV disease which include fatigue, weakness, weight gain, metabolic alterations, depression, cognitive impairment, cold intolerance, dry skin, and constipation [8,14,21,25,27]. Healthcare providers should be aware of these symptoms because untreated hypothyroidism makes HIV-related conditions worse while damaging patients’ quality of life and causing treatment noncompliance [8,9]. There is a need for vigilance for thyroid dysfunction when evaluating patients who experience unexplained fatigue or weight changes because of overlapping symptoms [5,9]. The health status of patients declines substantially when they develop overt hypothyroidism because it worsens fatigue symptoms, depression, and cognitive impairment, leading to decreased overall well-being [9,12,13].

The symptoms of overt hypothyroidism also include anaemia, bradycardia, peripheral neuropathy, and dyslipidemia. As discussed before, SCH can lead to cardiovascular complications. Women may experience infertility because of elevated prolactin levels and suppressed gonadotropin secretion, but thyroid hormone replacement therapy usually resolves this condition [20,46,47].

### 4.5. Screening for Overt Hypothyroidism in PLWH

The increased rate of overt hypothyroidism among PLWH requires doctors to perform standard thyroid function tests [8]. PLWH who receive cART treatment or have low CD4 counts may benefit from TSH and FT4 tests performed on a regular basis to detect thyroid problems early, though no current guidelines recommend routine monitoring for stable asymptomatic patients [8,9,15]. Early detection enables healthcare providers to address problems which prevents severe consequences [8]. The creation of clinical guidelines which integrate endocrinology professionals into patient care may be important for delivering optimal treatment [25]. The knowledge of hypothyroidism development after starting cART and IRIS becomes essential for better patient outcomes [31,41]. The screening interval is based on clinician judgement and symptoms development; however, this may only be once every 3 years. It is important to mention that there are no guidelines for screening frequency.

### 4.6. Diagnosis and Management

Medical tests reveal overt hypothyroidism by showing elevated TSH levels combined with low FT4 values [8,15,16,21,27]. Medical confirmation of diagnosis occurs through laboratory test results in combination with patient history and symptoms [8,25]. Testing for thyroid antibodies serves to reveal autoimmune origins in certain instances [27]. Levothyroxine serves as the primary treatment for hypothyroidism, while healthcare providers determine individual dosages through TSH measurement and patient feedback [14,15,16]. The patient needs continuous thyroid function test monitoring for cART changes, and achievement of hormone replacement. Monitoring can also aid the detection of drug interactions that occur when protease inhibitors reduce levothyroxine absorption [5,26]. The clinician needs to distinguish between HIV disease progression symptoms and thyroid disorders to achieve good outcomes [5,9]. The treatment of thyroid dysfunction in this population needs coordination between HIV specialists and endocrinologists and primary care providers for comprehensive patient care [10].

Standard levothyroxine treatment of HIV-positive patients with subclinical and overt hypothyroidism shows good results when patients receive proper monitoring. The use of protease inhibitors leads to decreased thyroxine bioavailability which necessitates changes in medication dosage. The risk of diastolic dysfunction with levothyroxine replacement may occur in PLWH which may require clinical assessment or imaging. The combination of endocrinologists and infectious disease specialists working together produces the best results while minimising the dangers of excessive or insufficient treatment [20,48].

## 5. Hyperthyroidism

Hyperthyroidism is a common thyroid disorder characterised by excess thyroid hormone (FT3 and/or FT4) production. By analysing hormone levels, hyperthyroidism can be classified as overt or subclinical. In overt hyperthyroidism, there is low or suppressed TSH levels, elevated FT3 levels and/or elevated FT4 levels. In subclinical hyperthyroidism, TSH is low or suppressed with normal FT3 and FT4 levels [49].

This disorder affects multiple organ systems physiologically. As a result, the signs and symptoms of hyperthyroidism are diverse. The manifestation of hyperthyroidism is often associated with a hyperadrenergic and hypermetabolic state. Some common signs and symptoms include unintentional weight loss, tremors, heat intolerance, palpitations, dyspnoea on exertion, irritability, anxiety, muscle weakness, increased frequency of bowel movements, hair loss, loss of libido, and oligomenorrhoea or amenorrhoea [49].

## 6. Overt Hyperthyroidism

Although overt hyperthyroidism is rare in PLWH, it is clinically significant. These patients can develop hyperthyroidism because of IRIS following the initiation of cART. The most common presentation is GD which is characterised by overt hyperthyroidism in the presence of thyroid-stimulating immunoglobulins (TSIs) [50]. There have been over 70 cases of hyperthyroidism reported that have presented as GD following cART therapy; however, this is likely underdiagnosed [51]. Among these cases, a Brazilian woman developed overt hyperthyroidism 45 months after cART (FT4: 28.9 pmol/L, TSH: <0.01 mlU/L, TRAb: 3.7 IU/L) [52]. Similarly, another patient in the U.S. developed classic GD symptoms of insomnia, exophthalmos, and heat intolerance two years following cART therapy. TSI levels for this patient were found to be >500% [41]. Many of the cases presented with autoimmune thyroid disease at a median of 8–33 months following cART [53,54]. Importantly, the median time for the development of hypothyroidism has not been reported in the literature. This time-period aligns with the time needed for immune restoration. The restoration can lead to IRIS through a rise in CD4+ T-cells, a shift toward pro-inflammatory cytokines, and a reactivation of autoreactive T-cell clones [51,55]. The pro-inflammatory cytokines are released by Th17 and Th1 cells. This causes the balance to shift toward more inflammation despite regulatory T-cell anti-inflammatory response. A subsequent increase in Th2 response may lead to autoantibody formation [50,55,56].

Studies have reported that up to 3% of women and 0.2% of men living with HIV may develop GD following cART [53,54]. This prevalence is 1.5–2 times higher than the general population. The risk may also be higher in African populations which may have a four-fold increase [53]. Furthermore, autoimmune thyroid disease can appear with other autoimmune conditions such as type 1 diabetes mellitus and myasthenia gravis because of immune reconstitution autoimmune disease (IRAD) [57,58]. 

Treatment for GD involves antithyroid medications such as methimazole and carbimazole [9,51,52,54,57,59]. Another strategy is to use radioactive iodine ablation (RAIT); however, one study evaluating response to RAIT found a 35.3% success rate at 3 months in patients with IRIS-related GD (IRIS-GD) compared to 63.4% in HIV-negative patients. Furthermore, adjusting for confounding factors showed that there was no difference in outcomes between the IRIS-GD patients and the HIV-negative patients in the short and long term [55].

Complications of GD have also presented as thyroid eye disease (TED) and pretibial myxoedema. TED is characterised by inflammation of muscles and orbital tissues, fibrosis, and deposition of glycosaminoglycans. Studies have shown that TED presents in 25% of GD cases with cART-induced IRIS being a risk factor. This is clinically significant as it may lead to permanent sight damage [41]. Another complication includes thyrotoxic periodic paralysis (TPP) which was reported in a case report [59]. This is a severe complication of GD and is characterised by acute flaccid paralysis and hypokalaemia. TPP occurred in an African American man that was taking cART for 8 years [59]. 

### Screening for Overt Hyperthyroidism in PLWH

There are no guidelines for hyperthyroidism screening in PLWH [51,52]. Evidence suggests that screening for all PLWH is not recommended but may be useful in certain cases. Due to the number of reports on IRIS-GD post-cART treatment, screening may be useful for patients that are taking cART, especially once the reconstitution of T-cells has been achieved. In addition, symptom-driven testing should be performed with vigilance for Graves’ disease [50,54].

## 7. Subclinical Hyperthyroidism

Although subclinical hyperthyroidism is more prevalent than overt hyperthyroidism, it is still uncommon. Some studies have reported a prevalence ranging from 0.5 to 2%, and some reported no cases [50,60]. A Nepalese study analysed a cohort of 203 HIV-positive patients, finding only 3 patients with subclinical hyperthyroidism (1.5%) [61]. In this study, there were no patients with overt hyperthyroidism. Another study conducted in Somalia analysed 101 HIV patients, reporting a prevalence for subclinical hyperthyroidism of 6.4% [62]. This study also found no cases of overt hyperthyroidism. Similarly, in Iran, another study found a prevalence for subclinical hyperthyroidism of 0.5% when analysing 209 patients [9]. 

Subclinical hyperthyroidism is often asymptomatic, resulting in incidental discovery through routine screening. Furthermore, it often self-resolves without the need for intervention. However, it may also progress to overt disease and is associated with cardiac risks, thus warranting clinical attention. Routine testing for subclinical hyperthyroidism is not recommended in asymptomatic PLWH unless there are symptoms of thyroid dysfunction or suspicion of immune reconstitution-related activity.

## 8. Thyroid Malignancy

Thyroid malignancy in PLWH is defined as an NADC. Other cancers that are also defined as NADCs include Hodgkin lymphoma and lung cancer. Interest in NADCs is increasing because of the rising trend in their prevalence when compared to AIDS-defining cancers (ADCs). The steady decline in ADCs since 2009 is thought to be due to the use of cART medications, earlier diagnosis, improved CD4+ T-cell recovery, and more effective HIV-1 viral load suppression [63]. 

Since 2010, the incidence of NADCs has risen which is ongoing to the present day. A study conducted in South Carolina found a higher prevalence of NADCs (4.4%) than ADCs (2.2%). The median time to diagnose was also longer for NADCs at 3.77 years compared to 1.95 years for ADCs. Furthermore, the study reported an association between NADCs and older age, co-infections, renal disease, hypothyroidism, and high-CD4+ T-cell count [63]. Another study conducted in Italy evaluating the prevalence of thyroid cancer in PLWH found a prevalence of 0.17% (*n* = 11) among a cohort of 6343 participants. Of these, papillary thyroid cancer (PTC) was most frequent at 63.63% (*n* = 7), followed by medullary thyroid cancer at 18.18% (*n* = 2), and follicular thyroid cancer at 9.1% (*n* = 1) [64]. More studies are needed to establish the prevalence of thyroid cancer in PLWH in different populations and to assess if cART and HIV are directly linked with thyroid cancer.

Mechanisms have been proposed, but further research is needed to verify these mechanisms. One study suggested that validation should be performed through in vivo or in vitro models [65]. Some mechanisms proposed include HIV causing chronic inflammation, impaired immune surveillance, hormonal axis disturbance, and oncogene and tumour suppressor gene dysregulation [22,65,66]. Overall, the current evidence suggests a strong association of thyroid cancer with HIV and cART; however, causation is yet to be established. Furthermore, cART has been associated with the development of autoimmune conditions, like GD, which can indirectly increase the risk of developing thyroid cancer [67]. 

Case studies have also reported more aggressive PTC in PLWH [67,68]. These patients presented with greater extrathyroid extension and lymph node involvement and staging [69]. This implies that immunosuppression may be associated with tumour progression [22,65] (Table 1). However, studies with larger cohorts should be conducted to verify this.

## 9. Conclusions

**Figure 2:**
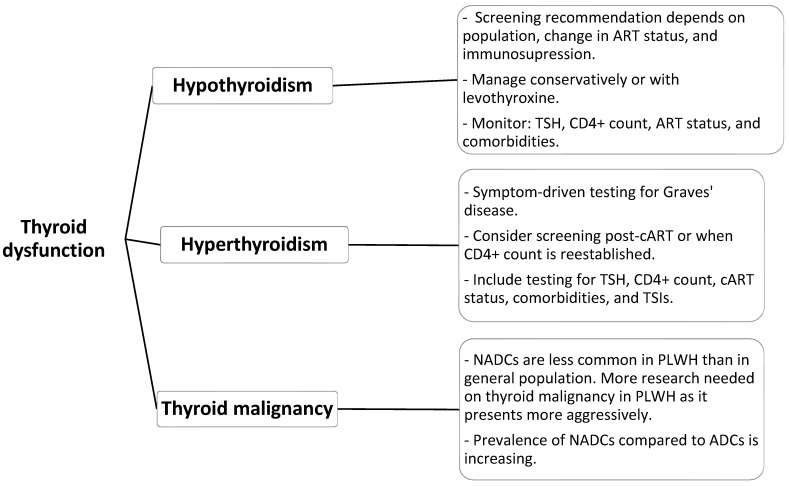
Proposed screening and investigations for thyroid dysfunction in PLWH.

Research acknowledges thyroid dysfunction as a major health condition affecting PLWH because of the extended survival rates resulting from effective cART regimens. The most common thyroid disorder found in people with low CD4 counts or long-term cART exposure is subclinical hypothyroidism, which can develop into overt disease if not detected. Although the occurrence of overt hyperthyroidism and GD remains rare, these conditions require clinical attention because they may develop from IRIS after cART initiation. The occurrence of thyroid malignancies remains infrequent, but these cancers seem to progress more aggressively within this population. The incorporation of endocrine monitoring into HIV management can lead to early discovery of thyroid conditions, enabling early treatment and better patient outcomes (Table 1) (Figure 2).

## Figures and Tables

**Table 1:** Summary of prevalences, key features, and screening recommendations for PLWH regarding thyroid dysfunction.

Type of Thyroid Dysfunction	Prevalence	Key Features	Screening Recommendation	Reference
Subclinical hypothyroidism	3.5–6.6%	High TSH, normal FT4; linked with cART and normal CD4+ count	Routine After change in cART status; with reduced CD4 count; with IRIS	[8]
Overt hypothyroidism	2.5–2.7%	High TSH, low FT4; linked to IRIS	Routine After change in cART status; with reduced CD4 count; with IRIS	[8]
Subclinical hyperthyroidism	0–6.4%	Low TSH, normal FT3/FT4, often asymptomatic	If symptomatic or IRIS is suspected	[50,60,62]
Overt hyperthyroidism	Male: 0.2% Female: 3%	Low TSH, high FT3/FT4; often TSI-positive; linked with post-cART IRIS; Graves’ disease most common	Symptom-based; consider screening post-cART and after immune reconstitution	[53]
Non-AIDS defining cancer-thyroid cancer	0.17%	More aggressive PTC with worse extrathyroid extension, lymph node involvement and staging	Clinical-based decision	[64,69]

## Data Availability

This review article and no new data generated.
